# Ultrasonographic Evaluation of Gastric Content and Volume in Pediatric Patients Undergoing Elective Surgery: A Prospective Observational Study

**DOI:** 10.3390/children10091432

**Published:** 2023-08-23

**Authors:** Asiye Demirel, Şeyda Efsun Özgünay, Şermin Eminoğlu, Ayşe Neslihan Balkaya, Tuğba Onur, Nermin Kılıçarslan, Mehmet Gamlı

**Affiliations:** Department of Anesthesiology and Reanimation, Bursa Yuksek Ihtisas Training and Research Hospital, University of Health Sciences, Bursa 16310, Turkey; seyda-efsun@hotmail.com (Ş.E.Ö.); sereminoglu1616@gmail.com (Ş.E.); aynesbalkaya@gmail.com (A.N.B.); doktor-t@hotmail.com (T.O.); nerminkilicarslan2001@gmail.com (N.K.); mehmetgamli@gmail.com (M.G.)

**Keywords:** aspiration in lungs, ultrasound examination of stomach, gastric antrum, gastric capacity, scheduled surgical procedures, child patients

## Abstract

Anesthesia-related complications, such as pulmonary aspiration of gastric contents, occur in approximately 0.02–0.1% of elective pediatric surgeries. Aspiration risk can be reliably assessed by ultrasound examination of the gastric antrum, making it an essential non-invasive bedside tool. In this prospective observational study, since most of our patients are immigrants and have communication problems, we wanted to investigate gastric contents and the occurrence of “high risk stomach” in children undergoing elective surgery for the possibility of pulmonary aspiration, even if the children and/or parents reported their last oral intake time. This risk is defined by ultrasound findings of solid content in the antrum and/or a calculated gastric volume exceeding 1.25 mL/kg. Children aged 2–18 were included in the study. Both supine and right lateral decubitus (RLD) ultrasound examinations were performed on the antrum before surgery. Using a qualitative grading scale from 0 to 2, we evaluated the gastric fluid content. The cross-sectional area (CSA) of the antrum was measured in the RLD position, aiding the calculation of the gastric fluid volume according to an established formula by Perlas. Ultrasound measurements of 97 children were evaluated. The median fasting duration was 4 h for liquids and 9 h for thick liquids and solids. Solid content was absent in all the children. Five children (5.2%) exhibited a grade 2 antrum, implying that fluid content was visible in both the supine and RLD positions. The median antral CSA in the RLD was 2.36 cm^2^, with a median gastric volume of 0.46 mL/kg. For patients with a grade 0 antrum, a moderate and positive correlation was observed between the antral CSA and BMI, and a strong and positive correlation was evident between the antral CSA and age, similar to a grade 1 antrum. Only a single child (1%) had a potentially elevated risk of aspiration of gastric contents. Hence, the occurrence of a “high risk stomach” was 1% (95% confidence interval: 0.1–4.7%) and is consistent with the literature. As a necessary precaution, we propose the regular use of ultrasound evaluations of gastric contents, given their non-invasive, bedside-friendly, and straightforward implementation, for identifying risks when fasting times are uncertain and for ruling out unknown risk factors in each potential patient.

## 1. Introduction

Pulmonary aspiration of gastric contents represents a complication in approximately 0.02–0.1% of elective pediatric surgeries [1]. This condition generally leads to anesthesia induction in patients with co-existing health issues or emergency surgery and often arises during tracheal extubation and recovery [2,3,4,5,6,7,8]. This can subsequently result in hypoxia, pneumonia, and extension of the mechanical ventilation requirements [9].

Gastric content volume, which, if increased, could lead to regurgitation and pulmonary aspiration, can be assessed using ultrasonography [10]. For gastric evaluation, bedside point of care ultrasound (POCUS) is a safe and non-invasive technique recommended by the European Society of Anesthesiology and Intensive Care [11,12]. This method enhances our understanding of gastric emptying in various patients and clinical circumstances, aiding in the identification of potential risk factors associated with gastric content [1,13,14]. Nil per os (NPO) guidelines are currently the standard preventive measures against aspiration. However, the inherent variability in gastric emptying among patients, co-existing health conditions, and unreliable NPO status pose potential risks [15]. The standard of care relies heavily on the patient’s or parents’ recall of the last oral intake, a practice vulnerable to inaccuracies and reliant on the communication abilities of the patient or parents. Time perception has been shown to be relatively weak, especially in the preoperative phase, and correlates with increased parental stress [16,17]. This calls into question the accuracy of the data, particularly in regions with high proportions of lower socioeconomic status groups, immigrants, and less effective communicators, where concerns about gastric content regurgitation and pulmonary aspiration in pediatric patients are prevalent.

In the past ten years, a number of groups, largely rooted in the realm of general anesthesia, have meticulously enhanced the utilization of POCUS in the appraisal of gastric contents [11,18,19,20,21,22,23,24,25,26,27,28,29]. This has been employed as an indirect measure for the risk of aspiration in the preparation phase of general anesthesia for adults, children, pregnant women, and the obese. A comprehensive model was first developed for adults by a Canadian group, spearheaded by Dr. Perlas [11,18,19,20,21]. The group designated the gastric antrum as a reliable imaging plane [11], crafted a qualitative ranking methodology based on visible content in the supine versus the right lateral decubitus (RLD) stances [18], and devised a model to project the gastric volume from the computed cross-sectional area (CSA) [11]. This model has been progressively sharpened and authenticated [19], leading to the model presently in use [20,21].

The existing pediatric framework was inferred from research conducted by Spencer et al. [24]. This study demonstrated a link between gastric ultrasound performed in the supine and RLD positions and the volumes aspirated endoscopically in pediatric patients lined up for endoscopy. A relationship between increased gastric volume and qualitative gastric grade was identified, along with a substantial correlation between the antral CSA and volume. This led to the creation of an equation to foresee the pediatric gastric volume from the CSA, akin to the adult model by Perlas.

In accordance with adult-oriented studies [11,18,19,22,23], guidelines have been developed to define a “high risk stomach” during the preparatory phase of general anesthesia. A “high risk stomach” is characterized by (1) the presence of any solid or thick fluid (considered “high risk”) or (2) clear liquid exceeding a specified volume limit (labeled as “indicative of high risk”) [20,21]. Ongoing debates in academic works concern the appropriate volume threshold to highlight cases at risk, with proposals ranging from 0.8 to 1.5 mL/kg [1,19,20,21,23,28,29]. The criteria for pediatric cases typically fall within a range of 1.2 to 1.5 mL/kg [24,30,31,32], substantially influenced by the study of Cook-Sather et al. [33]. This research, involving the gastric suctioning of 611 pediatric patients scheduled for elective surgery, determined that 95% of the subjects had a volume under 1.25 mL/kg. When the volume is not specifically measured, both adult [19] and child-focused studies [24] advocate for risk stratification based on qualitative grading.

This investigation aimed to evaluate the incidence of a “high risk stomach” characterized by ultrasound identification of solid matter and/or an estimated gastric fluid volume exceeding 1.25 mL/kg in elective procedures. Further, the research intended to contrast this characterization with a 0–2 qualitative grading scale, employed to distinguish between “empty” and “high risk stomach” conditions.

## 2. Materials and Methods

### 2.1. Research Framework

This prospective observational study, conducted from February 2020 to November 2021, obtained parental and/or patient informed consent and approval from the Ethics Committee of the University of Health Sciences, Bursa Yuksek Ihtisas Training and Research Hospital (Approval code: 2011-KAEK-25 2019/12-09, Approval Date: 25/12/2019). Data such as age, gender, weight, height, medical/surgical history, medications and their expiry times, allergies, and the last time clear liquids, thick liquids, and solid foods were consumed were collected.

### 2.2. Participant Selection

We examined a group of 100 participants, all ASA I-II, aged between 2 and 18 years, who were slated for elective surgical procedures under general anesthesia. Exclusion criteria were any previous gastrointestinal conditions, functional disorders, prior surgeries of the esophagus or upper abdomen, and inability to adopt the RLD position essential for gastric POCUS measurements.

Within our facility, it is customary for children set for elective procedures to consume clear liquids up to two hours ahead of anesthesia. They can have breast milk up to four hours beforehand, and milk, formula, or light meals are allowed up to six hours in advance, adhering to the prevalent guidelines [34].

The children underwent a range of procedures. These included pediatric surgical interventions like inguinal hernia repairs, circumcisions, pulmonary lobectomies, ingrown toenail removals, diagnostic laparoscopy, hysteroscopy, hypospadias corrections, pilonidal sinus treatments, cervical lymph node extractions, cystoscopies, meatotomies, scrotal cyst removals, treatments for undescended testes, and thyroglossal cyst removals. They also underwent urogenital surgeries such as circumcisions, orthopedic procedures to address humerus and femur fractures, and Ear Nose Throat (ENT) surgeries, including cervical lymph node removal and auricular mass extractions.

### 2.3. Gastric Antrum Ultrasound Examination

All ultrasonographic assessments of the gastric antrum were conducted by a single investigator, Dr. Asiye Demirel, an anesthesiologist possessing a professional background of 13 years and expertise in more than 100 gastric ultrasound evaluations. These evaluations were executed without knowledge of the patients’ clinical histories and were performed in the operating room right before the initiation of general anesthesia. The patients were first positioned supine and subsequently shifted to the RLD position. Depending on the distance of the antrum from the skin surface, either a low-frequency (2–5 MHz) convex abdominal probe or a high-frequency linear probe (5–12 MHz) was employed, adhering to previously established protocols [24,35].

Dr. Demirel conducted a qualitative assessment of the gastric antrum content utilizing the 3-point grading system as proposed by Perlas et al. [18]. The ratings were categorized as grade 0 (absence of content in a flattened antrum in both positions), grade 1 (fluid content visible only in the right lateral decubitus position), and grade 2 (fluid content noticeable in both the RLD and supine positions).

The antrum’s maximum anteroposterior (D1) and longitudinal diameters (D2) were gauged from serosa to serosa in the RLD orientation, and the antral cross-sectional area (CSA) was deduced using the following formula: antral CSA (cm^2^) = (π × D1 × D2)/4 [22]. Subsequently, the gastric volume was estimated through a previously validated mathematical formula specific to children: volume (mL·kg^(−1)^) = −7.8 + (3.5 × CSA (cm^2^)) + (0.127 × age {months})/body weight (kg) [24]. This model demonstrated an R^2^ value of 0.60 [24]. Assessments were suspended during any peristaltic motions of the antrum.

Stomach were classified as “high risk” if there was evidence of any solid, echogenic content in the antrum and/or if the gastric fluid volume > 1.25 mL/kg [24,33] and as “full” if any solid, echogenic content was in the antrum and/or a deduced gastric fluid volume of >1.5 mL/kg was noted [20,35]. A threshold of >1.25 mL/kg has been recommended for the identification of a “risky stomach” in pediatric cases [24,33].

In light of the indeterminate division between normal, upper normal, and augmented gastric volume, this investigation also ascertained the occurrence of children with solid content in the antrum and/or calculated gastric fluid volume of >0.8, >1, or >1.5 mL/kg. Such gastric volume cut-off values have been put forth to distinguish between an “empty” and “high risk stomach” in both adult and pediatric populations [13,18,20,23,30,31]. Gastric evaluations for each child were concluded within a time frame not exceeding 5 min.

Further, demographic information, timelines of solid food and liquid (clear and thick) consumption, surgical procedure type, and complications such as regurgitation and pulmonary aspiration were meticulously documented.

### 2.4. Sample Size and Statistical Assessment

Descriptive statistics are presented as numbers and percentages, whereas categorical data are presented as mean ± standard deviation, interquartile range, and minimum-maximum values. Fisher’s test was used to compare categorical data. The Kolmogorov–Smirnov test and histogram graphs were used to examine the assumption of a normal distribution. Since the data did not demonstrate normal distribution, the Friedman test was used for data comparison, and the Kruskal–Wallis test was used for inter-group analyses. Spearman correlation analysis was used to examine the correlation between data. Statistical significance was set at *p* < 0.05. The Bonferroni correction was applied for the *p*-value in the post hoc analyses. All analyses were performed using the SPSS 20.

Although no power analysis was performed at the start of the study, a post hoc power analysis was conducted to determine whether the study sample size was adequate during the analysis. The calculated effect sizes from the mean antral CSA and gastric volume values measured in the RLD position for all three grades were as follows: with an effect value of F = 1.49, F = 0.93, and a 5% alpha error value, the study power was over 99.9% in all analyses with 97 participants. The G*Power 3.1 Program performed for the analyses.

## 3. Results

**Figure 1:**
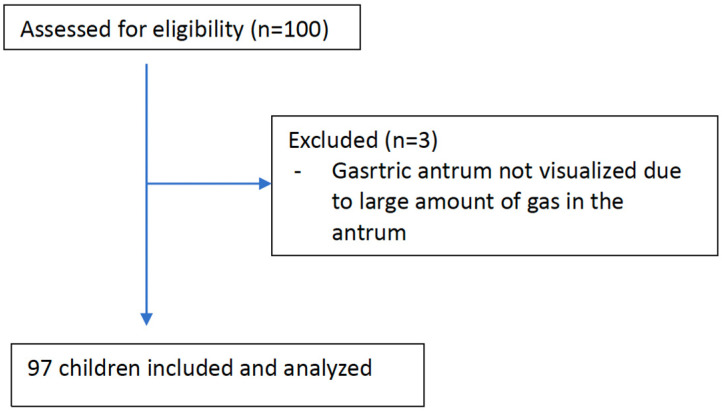
Flow diagram showing patient enrolment and analysis.

A total of 100 patients were assessed for eligibility, of which three were excluded due to excessive gas in the antrum, which obstructed sufficient evaluation. The analysis was carried out on 97 children who had definitive ultrasound examination results for the gastric antrum (Figure 1).

The preoperative evaluation and baseline characteristics are shown in Table 1.

The median (interquartile range) antral CSA was 2.36 (1.44–4.20) cm^2^ in the RLD position. The corresponding median gastric volume was 0.46 (0.33–0.72) mL/kg, and the median gastric volume was 13.41 (7.68–20.79) mL (Table 2). The median fasting time was 9 h for the solid/thick liquids and 4 h for the clear liquids. The last solid food intake was prolonged to 12 h (Table 2).

The correlation between the qualitative grading of the antrum from 0 to 2 and the antral CSA measured in the RLD position, alongside the associated gastric fluid volume, is outlined in Table 3. Notably, children with a grade 1 and grade 2 antrum demonstrated a significantly greater per-unit weight gastric fluid volume than those with a grade 0 antrum (Table 3).

Table 4 illustrates the distribution of children with calculated gastric fluid volumes exceeding 0.8, 1, 1.25, and 1.5 mL/kg, broken down by antrum grading. For a child with a grade 0 antrum and gastric fluid volume exceeding 0.8 mL/kg, the gastric fluid volume was still less than 1 mL/kg. Only one child had a gastric fluid volume exceeding 1.25 mL/kg (Table 4). This particular child, classified as ASA I, was slated for laparoscopic inguinal hernia surgery, with a 10 h fasting period for solid food and 2 h for clear liquids before ultrasound measurement. The prevalence of a “high risk stomach” in the study group was 1% (95% CI: 0.1–4.7%). The occurrence rates of gastric fluid volumes exceeding 0.8, 1.0, and 1.5 mL/kg were 19.6% (95% CI: 12.6–28.3%), 6.2% (95% CI: 2.6–12.3%), and 0.0%, respectively (Table 4). 

Among patients with a grade 0 antrum, a moderate positive correlation (Rho: 0.542, *p* < 0.001) was observed between the antral CSA in the RLD position and body mass index (BMI), as well as a strong positive correlation between the antral RLD CSA and age (Rho: 0.796, *p* < 0.001) (Table 5). For patients with a grade 1 antrum, there was a strong positive correlation between the antral RLD CSA and age (Rho: 0.622, *p* < 0.001) (Table 5). No case of regurgitation or pulmonary aspiration was noted.

## 4. Discussion

In our study, 97 children were assessed using ultrasound, and we discovered a grade 2 antrum in 5.2% of these participants in whom we were not sure of their fasting time and thought that their communication was incomplete. Only one child had a gastric volume greater than 1.25 mL/kg, indicating a “high-risk stomach” associated with an increased likelihood of pulmonary aspiration of gastric content. This means a mere 1% of children set for elective surgery exhibited gastric fluid volumes above 1.25 mL/kg, within a 95% confidence interval of 0.1–4.7% [20]. Ultimately, this is a very low percentage, but when pulmonary aspiration develops it can have serious consequences.

Previously conducted research found the mean gastric fluid volume to match the volumes aspirated blindly in children ready for elective surgery and the volumes determined in healthy volunteers through magnetic resonance imaging [33,36,37]. Another study that used a three-position blind aspiration method found the average gastric fluid volume to be 0.4 ± 0.45 mL/kg, with 95% of those children having volumes below 1.25 mL/kg [23]. On the other hand, 46% of children in emergency surgery had a higher volume of gastric contents assessed by nasogastric aspiration [33]. In our research, the proportion of children with a gastric fluid volume above 1.25 mL/kg, ranging from 0.1–4.7%, was lower compared to those exceeding 0.8 and 1 mL/kg. These findings reinforce the idea that surpassing 1.25 mL/kg of gastric fluid volume is rare in children who have fasted before elective surgery and may serve as a significant threshold to differentiate normal from increased gastric fluid volume, consistent with earlier studies [24,33]. 

Fortunately, the complexity of this complication extends beyond mere gastric content volume; the occurrence of pulmonary aspiration of gastric contents in children is notably below 1% and even less than 0.5% in certain instances [2,3]. Other elements, such as gastric inflation due to air insufflation during anesthesia induction, complications in managing the airway, and contractions or coughing linked to improper anesthesia techniques, also play roles in the development of pulmonary aspiration of gastric contents [4]. However, some investigations suggest that aspiration risk may rise independently of these factors [2,3]. 

In our study, the “high-risk stomach” classification (>1.25 mL/kg) applied to just one child, a six-year-old girl with a BMI of 13.22 kg/m^2^, prepared for ASA I and laparoscopic inguinal hernia surgery. She observed a 10 h fasting period for solid food and 2 h for clear liquids before the ultrasound evaluation. Even in the absence of apparent risk factors, her gastric volume was classified as “high-risk”. The literature points to an incremental growth in overall gastric volume with a constant RLD CSA as age progresses [24]. For example, a four-year-old child weighing 17 kg with an RLD CSA of 4 cm^2^ would have an estimated total gastric volume of 12 mL (0.7 mL/kg^−1^), while a 10-year-old child weighing 29 kg would have an estimated 21 mL [24]. Our study revealed a moderate positive correlation between the antral CSA and BMI for a grade 0 antrum and a strong positive correlation with age. Additionally, we identified a strong positive connection between the antral CSA and age for those with a grade 1 antrum. In alignment with different mathematical approaches [24,26,27,38], we chose to measure the antral CSA in the RLD position to hasten measurement completion, considering previous studies performed measurements in both the supine and RLD positions.

To focus on the elective pediatric population, where preoperative ultrasound examination of gastric contents could prove most valuable, additional studies should be performed to identify the risk factors that contribute to elevated gastric content volume. In our study, we found that 5.2% of children had a grade 2 antrum. This is similar to the findings of Desranges et al. and Spencer et al., who reported the incidence of a grade 2 antrum as 9.1% and 9%, respectively, in children scheduled for elective upper gastrointestinal endoscopy [24,31]. This higher rate can be attributed to the inclusion of children with gastrointestinal conditions such as inflammatory bowel disease or gastroesophageal reflux in the latter study [24]. Although our findings suggest that the use of the 0–2 qualitative rating scale may not be precise enough to differentiate between a “high risk stomach” and an “empty stomach” in children, they support the incidence of elective patients with a grade 2 antrum ranging between 3% and 5% [13,18]. The rate of risky stomachs identified in our study was consistent with that reported in the existing literature.

Furthermore, our study recorded fasting periods that were significantly longer than those recommended by current guidelines, which is common in most clinical practices [24,39,40]. This extended fasting could be attributed to slow changes in practice or scheduling constraints that prevent early morning procedures. Recent recommendations from various pediatric anesthesia societies suggest shorter fasting periods where feasible [41,42,43]. Current fasting time protocols have shown a good safety record in terms of low aspiration and regurgitation rates but often lead to excessive fasting durations, causing thirst and discomfort [40]. Moreover, several studies have demonstrated that the 2 h clear liquid fasting rule often results in real-world fasting times ranging from 6 to 13 h [44,45]. In our study, the median clear liquid fasting duration was 4 h and the median solid food fasting time was 9 h. Contrary to expectations, extending the fasting period does not reduce gastric fluid volume; instead, prolonged thirst and fasting can lead to significant preoperative agitation, perioperative hypotension, and ketone body accumulation [40,46,47,48]. Regardless of the fasting protocol or actual fasting duration, it should be noted that the risk of regurgitation or aspiration is associated with any sedation/general anesthetic procedure.

In recent studies, the effectiveness of imaging complete cross-sections via ultrasonography has been shown to be reduced when the stomach is filled with solid food [11]. While high correlation coefficients have been noted in various mathematical models to predict gastric volumes, with adjusted R^2^ values ranging from 0.60 to 0.73, these models have been found to be less reliable in cases of organic dyspepsia and gastroparesis [49]. This has spurred the development of computational simulations and modeling of gastric flow, offering valuable insights through integrative and quantitative in silico studies that may be challenging to perform experimentally [50,51,52]. Recent advancements in software technology have spurred the evolution of finite element-based computational simulation, making traditional mathematical formulas less sought-after. This computational simulation approach has emerged as an economical, timely, and more accessible method for investigating various intricate conditions that necessitate extensive resources and multifarious parameters [53]. Although the numerical simulation of the digestive system remains a nascent field, its potential for providing an enhanced understanding of gastrointestinal physiology and associated digestive ailments is being increasingly recognized. It holds promise as a robust methodology that could inform the development of novel diagnostic procedures and therapeutic interventions [53]. The prospects of creating a coupled multiphysics model of the human stomach would facilitate intricate in silico investigations into the digestion process, both in normal and pathological conditions [54]. These promising avenues are projected to stimulate rapid growth in research efforts over the forthcoming years, specifically focusing on the mathematical and computational modeling of gastric function [54].

### Limitations

Our study was conducted at a single center, limiting its generalizability and necessitating an expanded sample size. The volume of gastric content was not measured directly but rather estimated using ultrasound. Given the elective nature of the surgeries involved, invasive approaches, such as the use of gastric tubes for pre-anesthetic gastric content measurement in children, are unfeasible. Furthermore, blind aspiration may not extract all the gastric contents, potentially leading to inaccurate measurements. Other non-invasive gastric volume measurement techniques, such as gastric tomodensitometry or magnetic resonance, are unsuitable for elective surgeries because of practical and ethical considerations. However, these methods are not definitive for determining the volume of the gastric content. An additional factor to consider is that all ultrasound assessments in this study were performed by a single examiner. Nonetheless, a substantial body of prior research [13,18,20,23,24,26,27] attests to the accuracy of ultrasound and antral CSA measurements in assessing gastric volume and content in healthy individuals and surgical patients, both adult and pediatric, with high intra- and inter-rater reliability [55].

## 5. Conclusions

The risk of pulmonary aspiration from a “high risk stomach”, one of the most severe and debilitating complications of general anesthesia, should never be underestimated, even in elective pediatric surgeries. This is especially true when there is uncertainty about the child’s last fasting period or when there is a lack of thorough understanding of the patient or their parents. Furthermore, we found that 1% (95% CI: 0.1–4.7%) of children scheduled for elective surgery had a “high risk stomach”, indicating an increased risk of gastric content regurgitation and pulmonary aspiration. Although the incidence of aspiration in children is very low, it is significant for clinical practice. Ultrasound evaluation of gastric content is easy to perform, non-invasive, and a feasible bedside tool. Bedside imaging and interpretation should become essential parts of the best practice guidelines. As proposed, such a classification is designed to support medical decision-making and help plan and/or modify general anesthesia procedures. Future large-scale, multicenter studies of children undergoing elective surgery are needed to better define the target population.

## Figures and Tables

**Table 1:** Preoperative evaluation of the patients.

		*n*	(%)
Gender	Female	30	(30.9)
	Male	67	(69.1)
ASA	I	88	(90.7)
	II	9	(9.3)
Surgery type	Orthopedic surgery	2	(2.1)
	Pediatric surgery	83	(85.6)
	ENT surgery	4	(4.1)
	Urologic surgery	8	(8.2)
Gastric content	Grade 0	55	(56.7)
	Grade 1	37	(38.1)
	Grade 2	5	(5.2)

ASA: American Society of Anesthesiology, ENT: Ear, nose, and throat.

**Table 2:** Demographic data, last clear/thick liquid and solid intake time, antral RLD CSA, and gastric volume of the patients.

	Median (Interquartile Range)	Range (Min–Max)
Age (month)	72.00 (47.00–135.00)	24–212
Height (cm)	116.00 (103.00–147.00)	1.32–180
Weight (kg)	22.00 (16.50–41.00)	12–76
BMI (kg/m^2^)	17.46 (15.80–19.09)	12.75–23.89
Last solid intake time (h)	9.00 (8.00–9.00)	6–12
Last thick liquid intake time (h)	9.00 (8.00–9.00)	4–12
Last clear liquid intake time (h)	4.00 (3.00–6.00)	2–10
Antral RLD CSA (cm^2^)	2.36 (1.44–4.20)	0.48–6.34
Gastric volume (mL/kg)	0.46 (0.33–0.72)	0.1–1.29
Gastric volume (mL)	13.41 (7.68–20.79)	2.16–38.1

BMI: Body Mass Index, RLD: Right lateral Decubitus, CSA: Cross-sectional area, SD: Standard Deviation.

**Table 3:** Antral RLD CSA and gastric volume calculation of the antrum.

	Grade 0 (*n* = 55)	Grade 1 (*n* = 37)	Grade 2 (*n* = 5)	*p* ^a^
Antral RLD CSA (cm^2^) median (IQR)	1.49 (0.94–2.08)	4.29 (3.66–4.73)	5.96 (4.59–6.02)	**<0.001**
Antral RLD CSA (cm^2^) mean (95% CI)	1.58 (1.39–1.77)	4.25 (3.94–4.57)	5.44 (4.27–6.60)	
Calculated gastric volume (mL/kg) median (IQR)	0.34 (0.20–0.40)	0.72 (0.56–0.83)	0.81 (0.81–1.01)	**<0.001**
Calculated gastric volume (mL/kg) mean (95% CI)	0.32 (0.29–0.36)	0.72 (0.64–0.80)	0.93 (0.71–1.15)	

RLD: Right lateral decubitus, CSA: Cross sectional area. ^a^ Kruskal–Wallis test data are expressed as median (IQR: Interquartile range) or mean (95% confidence) intervals.

**Table 4:** The proportion of children with a calculated gastric fluid volume exceeding 0.8, 1, 1.25, and 1.5 mL/kg according to the grading of the antrum (*n* [%]).

	Grade 0 (*n* = 55)		Grade 1 (*n* = 37)		Grade 2 (*n* = 5)		*p* ^a^
Gastric volume > 0.8 mL/kg	1	(1.8%)	13	(35.1%)	5	(100.0%)	**<0.001**
Gastric volume > 1 mL/kg	0	(0.0%)	4	(10.8%)	2	(40.0%)	**0.002**
Gastric volume > 1.25 mL/kg	0	(0.0%)	1	(2.7%)	0	(0.0%)	0.433
Gastric volume > 1.5 mL/kg	0	(0.0%)	0	(0.0%)	0	(0.0%)	*

^a^ Fisher test, * *p* value could not be calculated due to lack of data.

**Table 5:** Correlation of antrum grade scores with BMI and age.

	Grade 0	Grade 1	Grade 2
	Rho (*p*)	Rho (*p*)	Rho (*p*)
BMI	0.542 (**<0.001**)	0.143 (0.397)	0.000 (>0.999)
Age (month)	0.796 (**<0.001**)	0.622 (**<0.001**)	0.667 (0.219)

BMI: Body mass index.

## Data Availability

Information and datasets used or produced during this research may be requested from the lead author, subject to reasonable conditions.

## References

[1] Bouvet L., Bellier N., Gagey-Riegel A.-C., Desgranges F.-P., Chassard D., Siqueira M.D.Q. (2018). Ultrasound assessment of the prevalence of increased gastric contents and volume in elective pediatric patients: A prospective cohort study. Pediatr. Anesth..

[2] Borland L.M., Sereika S.M., Woelfel S.K., Saitz E.W., Carrillo P.A., Lupin J.L., Motoyama E.K. (1998). Pulmonary aspiration in pediatric patients during general anesthesia: Incidence and outcome. J. Clin. Anesth..

[3] Tan Z., Lee S.Y. (2016). Pulmonary aspiration under GA: A 13-year audit in a tertiary pediatric unit. Pediatr. Anesth..

[4] Walker R.W. (2013). Pulmonary aspiration in pediatric anesthetic practice in the UK: A prospective survey of specialist pediatric centers over a one-year period. Pediatr. Anesth..

[5] Cook T.M., Woodall N., Frerk C., Fourth National Audit Project (2015). Major complications of airway management in the UK: Results of the Fourth National Audit Project of the Royal College of Anaesthetists and the Difficult Airway Society. Part 1 Anaesth..

[6] Cook T.M., Frerk C., Cook T.M., Woodall N., Frerk C. (2011). Chapter 19: Aspiration of gastric contents and of blood. Fourth National Audit Project of the Royal College of Anaesthetists and Difficult Airway Society.

[7] Warner M.A., Warner M.E., Warner D.O., Warner L.O., Warner E.J. (1999). Perioperative Pulmonary Aspiration in Infants and Children. Surv. Anesthesiol..

[8] Tiret L., Nivoche Y., Hatton F., Desmonts J.M., Vourc’H G. (1988). Complications related to anaesthesia in infants and children. Br. J. Anaesth..

[9] Habre W., Disma N., Virág K., Becke K., Hansen T.G., Jöhr M., Leva B., Morton N.S., Vermeulen P.M., Zielinska M. (2017). Incidence of severe critical events in paediatric anaesthesia (APRICOT): A prospective multicentre observational study in 261 hospitals in Europe. Lancet Respir. Med..

[10] Engelhardt T., Webster N.R. (1999). Pulmonary aspiration of gastric contents in anaesthesia. Br. J. Anaesth..

[11] Perlas A., Chan V.W.S., Lupu C.M., Mitsakakis N., Hanbidge A. (2009). Ultrasound Assessment of Gastric Content and Volume. Anesthesiology.

[12] Frykholm P., Disma N., Andersson H., Beck C., Bouvet L., Cercueil E., Elliott E., Hofmann J., Isserman R., Klaucane A. (2022). Pre-operative fasting in children. Eur. J. Anaesthesiol..

[13] Bouvet L., Desgranges F.-P., Aubergy C., Boselli E., Dupont G., Allaouchiche B., Chassard D. (2017). Prevalence and factors predictive of full stomach in elective and emergency surgical patients: A prospective cohort study. Br. J. Anaesth..

[14] Evain J.-N., Durand Z., Dilworth K., Sintzel S., Courvoisier A., Mortamet G., Desgranges F.-P., Bouvet L., Payen J.-F. (2021). Assessing gastric contents in children before general anesthesia for acute extremity fracture: An ultrasound observational cohort study. J. Clin. Anesth..

[15] Boretsky K.R., Perlas A. (2019). Gastric Ultrasound Imaging to Direct Perioperative Care in Pediatric Patients: A Report of 2 Cases. A A Pract..

[16] Grover G., Berkowitz C.D., Lewis R.J. (1994). Parental Recall After a Visit to the Emergency Department. Clin. Pediatr..

[17] Kelly C., Shulman V., Khine H., Avner J.R. (2007). Parental Perception of the Passage of Time During a Stressful Event. Pediatr. Emerg. Care.

[18] Perlas A., Davis L., Khan M., Mitsakakis N., Chan V.W. (2011). Gastric sonography in the fasted surgical patient: A prospective descriptive study. Anesth. Analg..

[19] Perlas A., Mitsakakis N., Liu L., Cino M., Haldipur N., Davis L., Cubillos J., Chan V. (2013). Validation of a Mathematical Model for Ultrasound Assessment of Gastric Volume by Gastroscopic Examination. Obstet. Anesth. Dig..

[20] Perlas A., Van de Putte P., Van Houwe P., Chan V.W.S. (2015). I-AIM framework for point-of-care gastric ultrasound. Br. J. Anaesth..

[21] Perlas A., Arzola C., Van de Putte P. (2018). Point-of-care gastric ultrasound and aspiration risk assessment: A narrative review. Can. J. Anaesth..

[22] Bouvet L., Miquel A., Chassard D., Boselli E., Allaouchiche B., Benhamou D. (2009). Could a single standardized ultrasonographic measurement of antral area be of interest for assessing gastric contents? A preliminary report. Eur. J. Anaesthesiol..

[23] Bouvet L., Mazoit J.-X., Chassard D., Allaouchiche B., Boselli E., Benhamou D. (2011). Clinical Assessment of the Ultrasonographic Measurement of Antral Area for Estimating Preoperative Gastric Content and Volume. Anesthesiology.

[24] Spencer A.O., Walker A.M., Yeung A.K., Lardner D.R., Yee K., Mulvey J.M., Perlas A. (2014). Ultrasound assessment of gastric volume in the fasted pediatric patient undergoing upper gastrointestinal endoscopy: Development of a predictive model using endoscopically suctioned volumes. Pediatr. Anesth..

[25] Moser J.J., Walker A.M., Spencer A.O. (2017). Point-of-care paediatric gastric sonography: Can antral cut-off values be used to diagnose an empty stomach?. Br. J. Anaesth..

[26] Schmitz A., Thomas S., Melanie F., Rabia L., Klaghofer R., Weiss M., Kellenberger C. (2011). Ultrasonographic gastric antral area and gastric contents volume in children. Pediatr. Anesth..

[27] Schmitz A., Schmidt A.R., Buehler P.K., Schraner T., Frühauf M., Weiss M., Klaghofer R., Kellenberger C.J. (2016). Gastric ultrasound as a preoperative bedside test for residual gastric contents volume in children. Pediatr. Anesth..

[28] Van de Putte P., Perlas A. (2014). Gastric sonography in the severely obese surgical patient: A feasibility study. Anesth. Analg..

[29] Kruisselbrink R., Arzola C., Jackson T., Okrainec A., Chan V., Perlas A. (2016). Ultrasound assessment of gastric volume in severely obese individuals: A validation study. Br. J. Anaesth..

[30] Van de Putte P., Perlas A. (2018). The link between gastric volume and aspiration risk. In search of the Holy Grail?. Anaesthesia.

[31] Desgranges F.-P., Riegel A.-C.G., Aubergy C., Siqueira M.D.Q., Chassard D., Bouvet L. (2017). Ultrasound assessment of gastric contents in children undergoing elective ear, nose and throat surgery: A prospective cohort study. Anaesthesia.

[32] Leviter J., Steele D.W., Constantine E., Linakis J.G., Amanullah S. (2019). “Full stomach” despite the wait: Point-of-care gastric ultrasound at the time of procedural sedation in the pediatric emergency department. Acad. Emerg. Med..

[33] Cook-Sather S.D., Liacouras C.A., Previte J.P., Markakis D.A., Schreiner M.S. (1997). Gastric fluid measurement by blind aspiration in paediatric patients: A gastroscopic evaluation. Can. J. Anaesth..

[34] (2017). Practice guidelines for preoperative fasting and the use of pharmacologic agents to reduce the risk of pulmonary aspiration: Application to healthy patients undergoing elective procedures: An updated report by the American Society of Anesthesiologists Task Force on Preoperative Fasting and the Use of Pharmacologic Agents to Reduce the Risk of Pulmonary Aspiration. Anesthesiology.

[35] Gagey A.-C., Siqueira M.d.Q., Desgranges F.-P., Combet S., Naulin C., Chassard D., Bouvet L. (2016). Ultrasound assessment of the gastric contents for the guidance of the anaesthetic strategy in infants with hypertrophic pyloric stenosis: A prospective cohort study. Br. J. Anaesth..

[36] Schmitz A., Kellenberger C.J., Liamlahi R., Fruehauf M., Klaghofer R., Weiss M. (2011). Residual gastric contents volume does not differ following 4 or 6 h fasting after a light breakfast—A magnetic resonance imaging investigation in healthy non-anaesthetised school-age children. Acta Anaesthesiol. Scand..

[37] Schmitz A., Kellenberger C., Lochbuehler N., Fruehauf M., Klaghofer R., Weiss M. (2012). Effect of different quantities of a sugared clear fluid on gastric emptying and residual volume in children: A crossover study using magnetic resonance imaging. Br. J. Anaesth..

[38] Gagey A.C., de Queiroz Siqueira M., Monard C., Combet S., Cogniat B., Desgranges F.-P., Robinson P., Chassard D., Bouvet L. (2018). The effect of pre-operative gastric ultrasound examination on the choice of general anaesthetic induction technique for non-elective pediatric surgery. A prospective cohort study. Anesthesia.

[39] Falconer R., Skouras C., Carter T., Greenway L., Paisley A.M. (2013). Preoperative fasting: Current practice and areas for improvement. Updat. Surg..

[40] Engelhardt T., Wilson G., Horne L., Weiss M., Schmitz A. (2011). Are you hungry? Are you thirsty?—Fasting times in elective outpatient pediatric patients. Pediatr. Anesth..

[41] Andersson H., Hellström P.M., Frykholm P. (2017). Introducing the 6-4-0 fasting regimen and the incidence of prolonged preoperative fasting in children. Pediatr. Anesth..

[42] Newton R.J.G., Stuart G.M., Willdridge D.J., Thomas M. (2017). Using quality improvement methods to reduce clear fluid fasting times in children on a preoperative ward. Pediatr. Anesth..

[43] Thomas M., Morrison C., Newton R., Schindler E. (2018). Consensus statement on clear fluids fasting for elective pediatric general anesthesia. Pediatr. Anesth..

[44] Williams C., Johnson P.A., Guzzetta C.E., Guzzetta P.C., Cohen I.T., Sill A.M., Vezina G., Cain S., Harris C., Murray J. (2014). Pediatric Fasting Times Before Surgical and Radiologic Procedures: Benchmarking Institutional Practices Against National Standards. J. Pediatr. Nurs..

[45] Al-Robeye A.M., Barnard A.N., Bew S. (2019). Thirsty work: Exploring children’s experiences of preoperative fasting. Pediatr. Anesth..

[46] Brady M.C., Kinn S., Ness V., O’Rourke K., Randhawa N., Stuart P. (2009). Preoperative fasting for pre-venting perioperative complications in children. Cochrane Database Syst. Rev..

[47] Dennhardt N., Beck C., Huber D., Sander B., Boehne M., Boethig D., Leffler A., Sümpelmann R. (2016). Optimized preoperative fasting times decrease ketone body concentration and stabilize mean arterial blood pressure during induction of anesthesia in children younger than 36 months: A prospective observational cohort study. Pediatr. Anesth..

[48] Simpao A.F., Wu L., Nelson O., Gálvez J.A., Tan J.M., Wasey J.O., Muhly W.T., Tsui F.-C., Masino A.J., Stricker P.A. (2020). Faculty Opinions recommendation of Preoperative fluid fasting times and postinduction low blood pressure in children: A retrospective analysis. Anesthesiology.

[49] Tan Y., Wang X., Yang H., Pan C., Luo N., Li J., Yang F., Bei Y., Cahilog Z., Chen Q. (2022). Ultrasonographic assessment of preoperative gastric volume in patients with dyspepsia: A prospective observational study. BMC Anesthesiol..

[50] Harrison S.M., Cleary P.W., Sinnott M.D. (2018). Investigating mixing and emptying for aqueous liquid content from the stomach using a coupled biomechanical-SPH model. Food Funct..

[51] Kozu H., Kobayashi I., Nakajima M., Uemura K., Sato S., Ichikawa S. (2010). Analysis of Flow Phenomena in Gastric Contents Induced by Human Gastric Peristalsis Using CFD. Food Biophys..

[52] Pal A., Brasseur J.G., Abrahamsson B. (2007). A stomach road or ‘Magenstrasse’ for gastric emptying. J. Biomech..

[53] Jamari J., Ammarullah M.I., Santoso G., Sugiharto S., Supriyono T., Permana M.S., Winarni T.I., van der Heide E. (2022). Adopted walking condition for computational simulation approach on bearing of hip joint prosthesis: Review over the past 30 years. Heliyon.

[54] Brandstaeter S., Fuchs S.L., Aydin R.C., Cyron C.J. (2019). Mechanics of the stomach: A review of an emerging field of biomechanics. Gamm-Mitteilungen.

[55] Kruisselbrink R., Arzola C., Endersby R., Tse C., Chan V., Perlas A. (2014). Intra- and Interrater Reliability of Ultrasound Assessment of Gastric Volume. Anesthesiology.

